# Vulnerability of Mediterranean Ecosystems to Long-Term Changes along the Coast of Israel

**DOI:** 10.1371/journal.pone.0102090

**Published:** 2014-07-08

**Authors:** David Kaniewski, Elise Van Campo, Christophe Morhange, Joël Guiot, Dov Zviely, Sabine Le Burel, Thierry Otto, Michal Artzy

**Affiliations:** 1 Laboratoire d’Ecologie Fonctionnelle et Environnement (EcoLab), Université Paul Sabatier-Toulouse 3, Toulouse, France; 2 Laboratoire d’Ecologie Fonctionnelle et Environnement (EcoLab), Centre National de la Recherche Scientifique, Toulouse, France; 3 Institut Universitaire de France, Paris, France; 4 Centre de Recherche et d’Enseignement de Géosciences de l’Environnement (CEREGE), Unité Mixte 34, Aix-Marseille Université, Centre National de la Recherche Scientifique, Aix-en-Provence, France; 5 Leon Recanati Institute for Maritime Studies, University of Haifa, Haifa, Israel; 6 Hatter Laboratory, University of Haifa, Haifa, Israel; Point Blue Conservation Science, United States of America

## Abstract

Although human activity is considered to be a major driving force affecting the distribution and dynamics of Mediterranean ecosystems, the full consequences of projected climate variability and relative sea-level changes on fragile coastal ecosystems for the next century are still unknown. It is unclear how these waterfront ecosystems can be sustained, as well as the services they provide, when relative sea-level rise and global warming are expected to exert even greater pressures in the near future (drought, habitat degradation and accelerated shoreline retreat). Haifa Bay, northern Israel, has recorded a landward sea invasion, with a maximum sea penetration 4,000 years ago, during an important period of urban development and climate instability. Here, we examine the cumulative pressure of climate shifts and relative sea-level changes in order to investigate the patterns and mechanisms behind forest replacement by an open-steppe. We provide a first comprehensive and integrative study for the southern Levant that shows that (i) human impact, through urbanization, has been the main driver behind ecological erosion in the past 4,000 years; (ii) climate pressures have reinforced this impact; and (iii) local coastal changes have played a decisive role in eroding ecosystem resilience. These three parameters, which have closely interacted during the last 4,000 years in Haifa Bay, clearly indicate that for an efficient management of the coastal habitats, anthropogenic pressures linked to urban development must be reduced in order to mitigate the predicted effects of Global Change.

## Introduction

Mitigating the impact of ongoing climate change and increasing human activities on ecosystems is one of the biggest challenges of the coming decades, at all geographical scales and across all economic spheres [1]–[5]. Coastal ecosystems are among the most threatened because relative sea-level rise associated with global warming renders coastal habitats particularly vulnerable [6]–[7]. High-resolution climate simulations conducted worldwide predict that temperature increases will severely affect the Eastern Mediterranean (EM) over the next century [8]–[10]. An intensification of extreme weather events is also predicted to interact with patterns of species distribution even if non-climatic influences may dominate local short-term biological shifts [11]. Moreover, regional patterns of warming-induced changes in hydrology seem to be more complex and less certain than those in temperature, chiefly in the EM’s seabord areas [8], [12]–[13]. Shoreline changes, which may result from a range of interacting physical and chemical processes, will also have important consequences for biological features of the EM coastlines [14]–[15].

**Figure 1:**
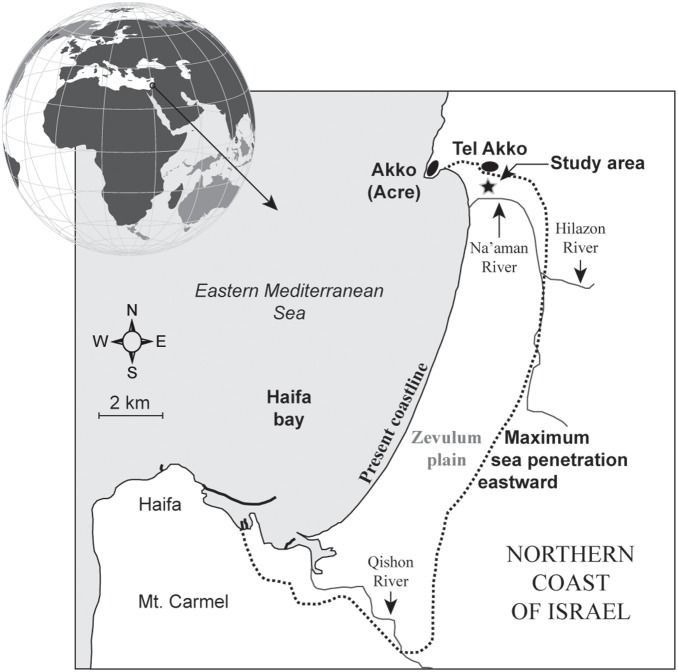
Map of Haifa Bay in northern Israel. The study site is denoted by a black star. The maximum marine ingression during the Bronze Age is shown by a dotted line, with the present coastline as reference.

Despite the importance of Mediterranean coastal vegetation in earth-atmosphere energy budgets [16] and ecosystem functioning [17], there is still a paucity of empirical data concerning long-term ecosystem distributions in the face of human impacts, climate pressures and coastline changes. This gap is most dramatic for the southeastern Mediterranean coasts, an outstanding center of biodiversity but also one of the most threatened, by economic activity, coastal modifications and drought [6]. Here we investigate 6000 years of coastal ecosystem dynamics in Haifa Bay (Fig. 1), the northernmost depositional sediment sink of the Nile littoral cell [18]. Pollen-derived information on vegetation changes from Akko (Acre), Akko Plain, was used to reconstruct biological trends and assess the role of the local landward sea invasion and subsequent coastal progradation in ecological erosion at the millennial/centennial scale. Previous research conducted at Akko has highlighted human impacts since the early phases of urban development [19]. Here, the data are used to decipher the relative pressures resulting from the sea ingression and climate shifts. The long-term changes along the coasts of Israel [7], [20] that have caused strong biological stresses and major ecological alterations during the past 6000 years, can be seen as a model to understand the consequences of Mediterranean relative sea-level rise for the next century (up to 61 cm) [14].

## Haifa Bay

The coastal part of Israel is subject to the basic pattern of Mediterranean climate with mild rainy winter and hot summers, with decreasing amount of rainfall and increasing temperature southward. At Akko, annual precipitation and mean temperature are 499 mm and 20°C, respectively. The Mediterranean forests of Israel historically comprised evergreen, sclerophyllous and pine tree species, which were destroyed over several millennia by ancient civilizations for agriculture, shipbuilding and fuel. Today the predominant species in the low altitude coastal woodlands are the carob (*Ceratonia siliqua*), Pistachio (*Pistacia lentiscus*), oak (*Quercus calliprinos*) and Aleppo pine (*Pinus halepensis*) [21]. In the Zevulun Plain, a rift valley between Haifa and Akko, underground flow of seawater inland and its mixing with freshwater enables the development of salt marshes populated by *Arthrocnemum macrostachyum, Sarcocornia fruticosa, Limonium narbonense, Atriplex portulacoides* and *Tamarix tetragyna* [21].

## Methods

### Core and modern samples

**Figure 2:**
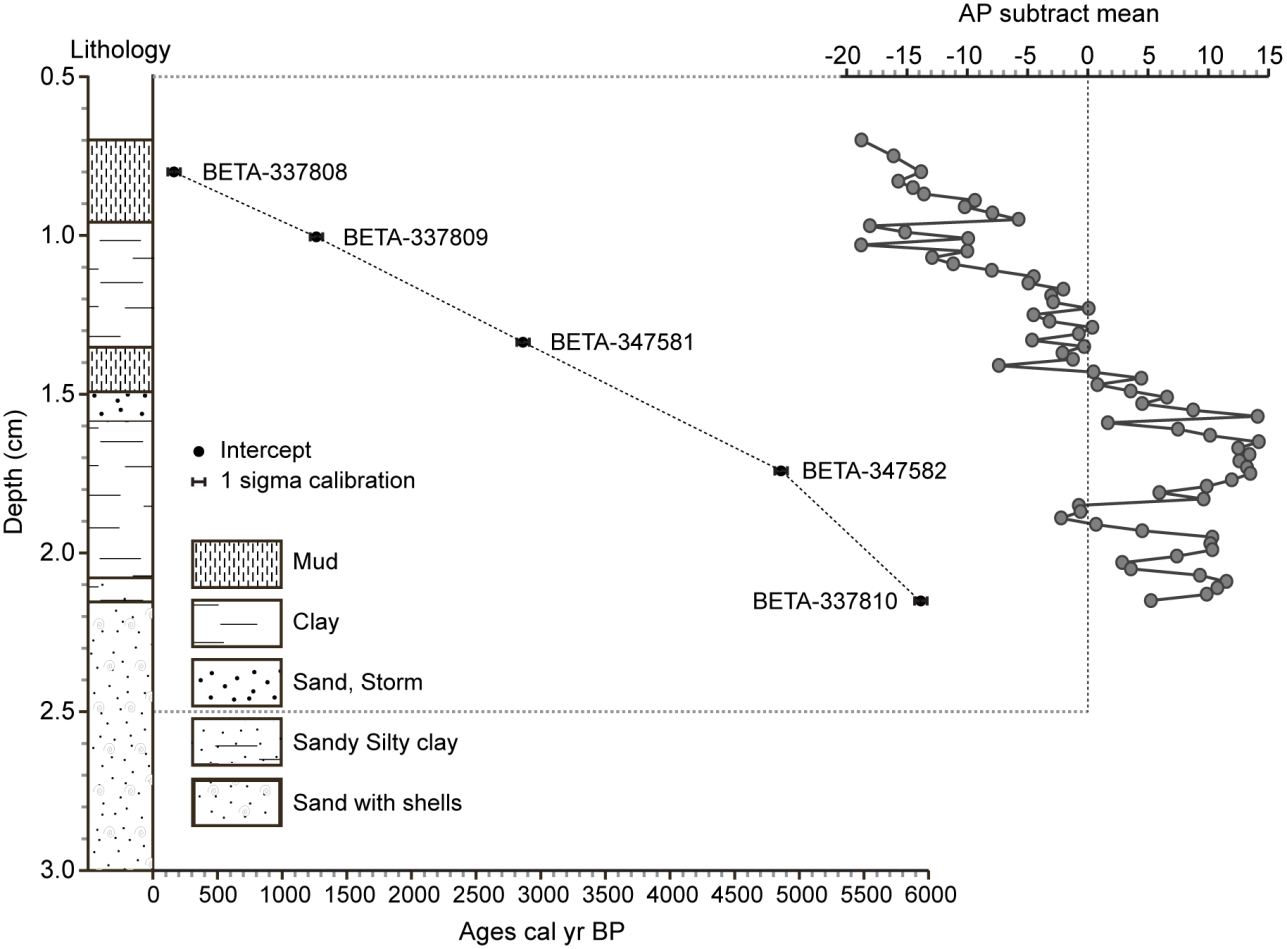
AMS ^14^C calibrated ages (1σ-68%) and suggested age-depth curve. The lithology of the core sampled at Akko is detailed and plotted on a linear depth-scale. The arboreal pollen curve from the core is shown as “subtract mean” function and plotted on a linear depth-scale.

**Figure 3:**
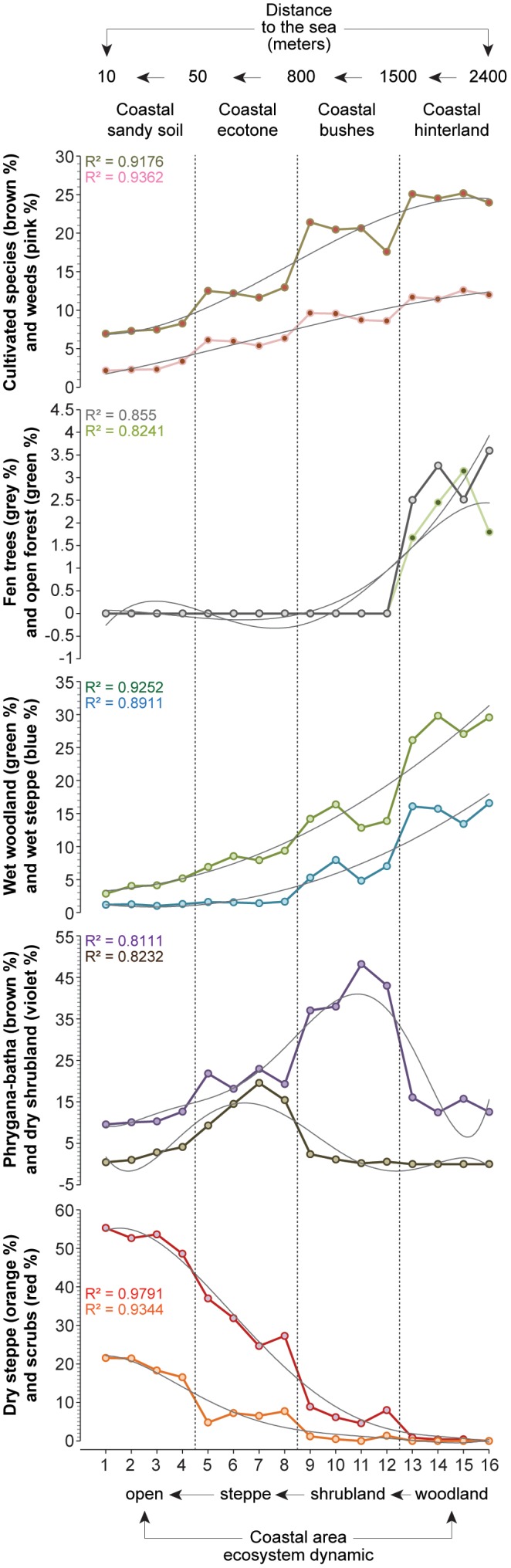
Modern pollen deposition from the northern Haifa Bay expressed as pollen-derived clusters. The polynomial curves indicate the general trend. A variety of vegetation types from the sandy shore (open steppe) to the coastal hinterland (woodland) were selected.

Biological indicators, extracted from a 215-cm continuous core (32°54′N, 35°05′E; +3 m above sea-level, about 600 m from the sea) drilled in the northern part of Haifa Bay, were used to reconstruct the long-term ecosystem dynamics and plant distribution in the coastal area. Work permits were obtained through to the University of Haifa (the Leon Recanati Institute for Maritime Studies and the Hatter Laboratory). The field studies did not involve endangered or protected species. The chronology is based on accelerator mass spectrometry (AMS) radiocarbon (^14^C) ages of short-lived terrestrial samples (seeds; Fig. 2). Modern plant species distributions (Fig. 3) were assessed using pollen deposition. Sample locales (Table S1) were chosen to cover a variety of vegetation types from the sandy shore (32°58′N, 35°04′E; +1 m above sea-level, about 10 m from the sea) to the coastal hinterland (32°54′N, 35°06′E; +8 m above sea-level, about 2.4 km from the sea; Table 1). They were also selected within the broad city limits, in order to ensure that vegetation communities were subjected to similar anthropogenic pressure and that the “distance to the sea” was their main source of ecological stress (inversely related to the distance). The distances to the sea for the modern samples are indicated in Figure 3 and in Table 1.

**Table 1:** Geographical details of the modern samples.

	Coordinates		Altitude	Distance to the sea
Modern samples	North	East	(m a.s.l.)	(m)
**1**	32°55′50.51″	35°04′12.99″	5	13
**2**	32°55′18.15″	35°04′20.88″	7	17
**3**	32°55′15.92″	35°04′31.05″	5	31
**4**	32°56′11.52″	35°04′22.43″	4	40
**5**	32°54′39.70″	35°04′54.80″	2	59
**6**	32°54′43.08″	35°04′59.63″	3	210
**7**	32°54′30.28″	35°05′14.73″	3	519
**8**	32°54′41.44″	35°05′23.18″	2	775
**9**	32°53′33.73″	35°05′14.02″	6	813
**10**	32°52′45.17″	35°05′12.96″	8	1138
**11**	32°51′50.38″	35°04′59.01″	10	1294
**12**	32°52′43.44″	35°05′26.49″	7	1493
**13**	32°57′20.58″	35°05′30.38″	12	1591
**14**	33°03′52.51″	35°07′25.82″	16	1828
**15**	33°00′20.61″	35°06′45.12″	20	2187
**16**	32°59′38.26″	35°06′42.90″	27	2512

The coordinates (°N, °E), the altitudes and the distances to the sea are given.

### Biological data

A total of 86 samples were prepared for pollen analysis using the standard palynological procedure for clay and surface samples [22]. Pollen grains and dinoflagellate cysts were counted under x400 and x1000 magnification. Pollen frequencies (%) are based on the terrestrial pollen sum excluding local hygrophytes and the spores of non-vascular cryptogams. The arboreal pollen was summed and is depicted as a single curve (using the “subtract mean” function) to provide visualisation of the deviation from the average value (Fig. 2). Dinoflagellate cysts were counted on pollen-slides and are displayed as concentrations (cysts per cm^−3^). The dinoflagellate cysts (resting stage of marine plankton), were used as an independent proxy for the sea incursion [23]. The biological data were resampled (AnalySeries 2.0; sampling grid: one sample every 50 years) in order to obtain an identical chronological scale for all the proxies.

### Multivariate analyses

Our aim is to integrate each sample from the core into a modern vegetation transect, based on its “*distance to the sea*”. Our goal is to study the vegetation dynamics during the last 6000 years, using present distributions as analogues. All numerical analyses were performed with the software PAST.

**Figure 4:**
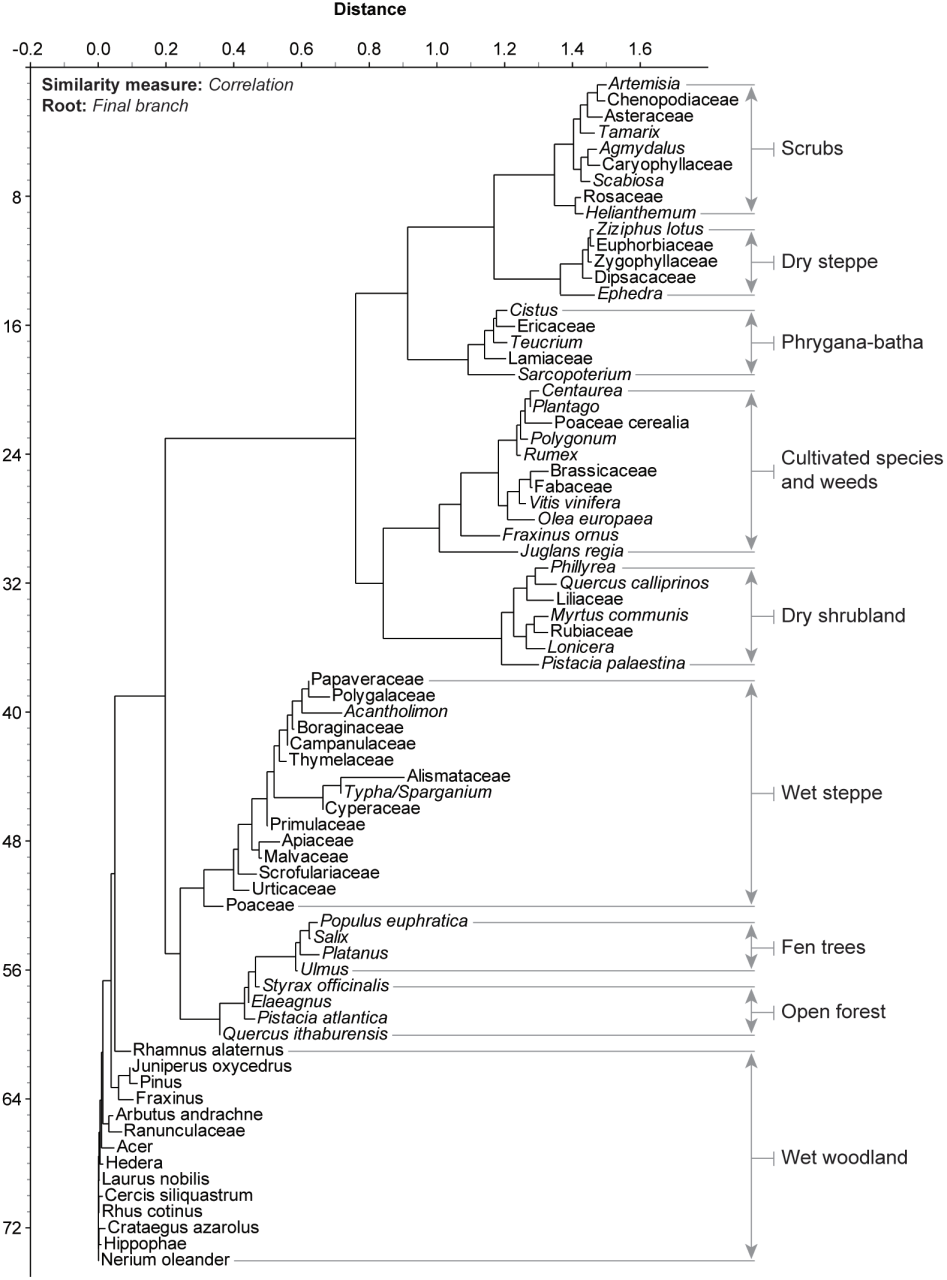
Neighbour Joining analysis of the main taxa (modern and fossil) computed with *correlation* as similarity measure and *final branch* as root. The pollen-types from each cluster were summed to create pollen-derived vegetation patterns.

All pollen data (fossil and modern) were transformed into pollen-derived clusters (^Pd^Clusters) using Neighbour Joining (NJ) analysis (Fig. 4). The NJ method is an alternative process for hierarchical cluster analysis, finding hierarchical groupings in multivariate data sets. Here, it is based on pollen-type time-series (presence/absence and abundance). NJ analysis was used to compute the lengths of branches of a tree, using branches as ecological distances between groups of taxa (descending type). NJ was computed using correlation as similarity measure and final branch as root. The pollen-types from each cluster were summed (Tables S1 and S2) to create local pollen-derived vegetation patterns (Figs 3 and 4).

**Figure 5:**
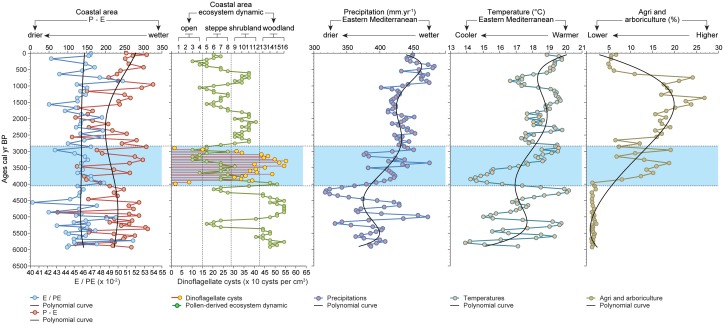
A 6000-year environmental reconstruction of Haifa Bay. P-E, E/PE, precipitation, temperatures and anthropogenic activities are displayed as scores, mm. yr^−1^, °C and %. A polynomial curve was established for each component and plotted on a linear timescale. The coastal ecosystem dynamics and the dinoflagellate cysts (marine phytoplankton) were also plotted on a linear timescale. The numbers associated with the coastal ecosystem dynamics are related to the modern samples (Fig. 3). The marine ingression is underlined in blue and is bracketed between horizontal dotted lines.

Each sample from the core, expressed as PdCluster-scores, was integrated in the modern pollen deposition matrix (also expressed as PdCluster-scores), and then assigned to a modern distribution using Principal Components Analysis (PCA). The method is based on the PCA axes 1 and 2 (that load the maximum amount of total variance) and only selects the closest score between one modern sample and the fossil sample. The fossil sample was, according to the outcome of the “closest score” method, affiliated to one modern sample. Each affiliation was controlled by cluster analysis (with paired group as algorithm and correlation as the similarity measure) and by k-means clustering (number of clusters: 16), using the same pollen deposition matrix as the PCA and testing the fossil samples one by one. The palaeo-distributions were then recompiled in one unique curve in order to create a proxy for vegetation dynamics (^P^VD) along the Mediterranean coast of Israel for the last 6000 years (Fig. 5).

**Figure 6:**
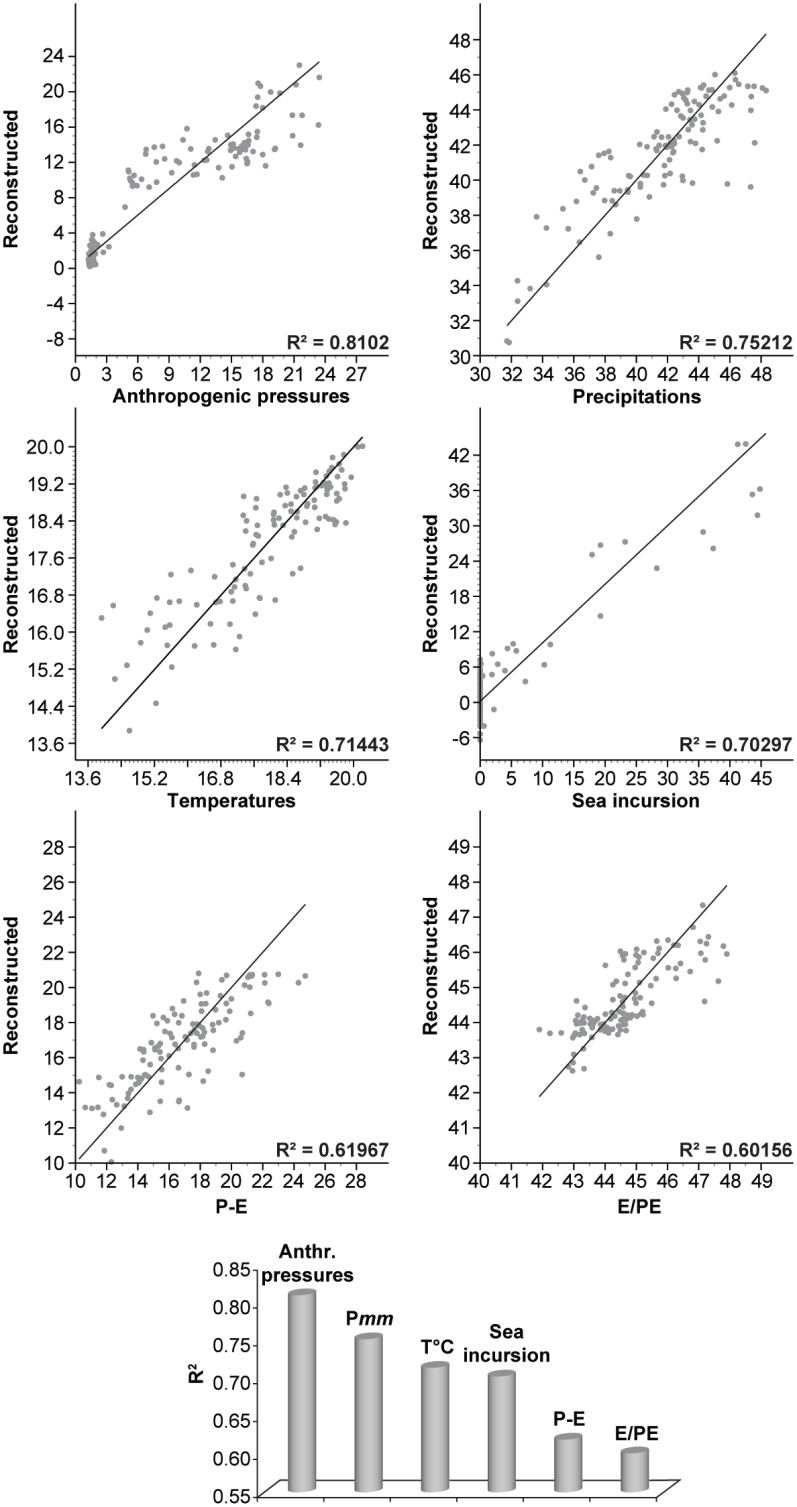
Environmental regression models from Haifa Bay. The selected environmental data are indicated below each graph. The consistency of each reconstructed model is indicated by the R^2^. The lower graph corresponds to a ranking of the environmental data according to the R^2^ values.

The ^Pd^Clusters were also analysed using CABFAC analysis (Fig. 6) to test the impact of environmental factors on the ^P^VD. The CABFAC factor analysis (Q-mode factor analysis) implements the classical method of factor analysis and environmental regression (CABFAC and REGRESS). Selected environmental data are regressed on the CABFAC factors using the second-order (parabolic) method, with cross terms. The environmental regression model (RMA) reports the observed values against the values reconstructed from the factors. The selected environmental data for this study [anthropogenic pressures; EM precipitation; EM temperature; sea incursion; ratio of actual and potential evapotranspiration (E/PE) for the EM; difference between annual precipitation and actual evapotranspiration (P-E) for the EM] cover a wide range of pressures. The consistency of each reconstructed model is indicated by the R^2^. The R^2^ values were ordered from the highest to the lowest, and used as a hierarchy of pressures on ecosystems.

**Figure 7:**
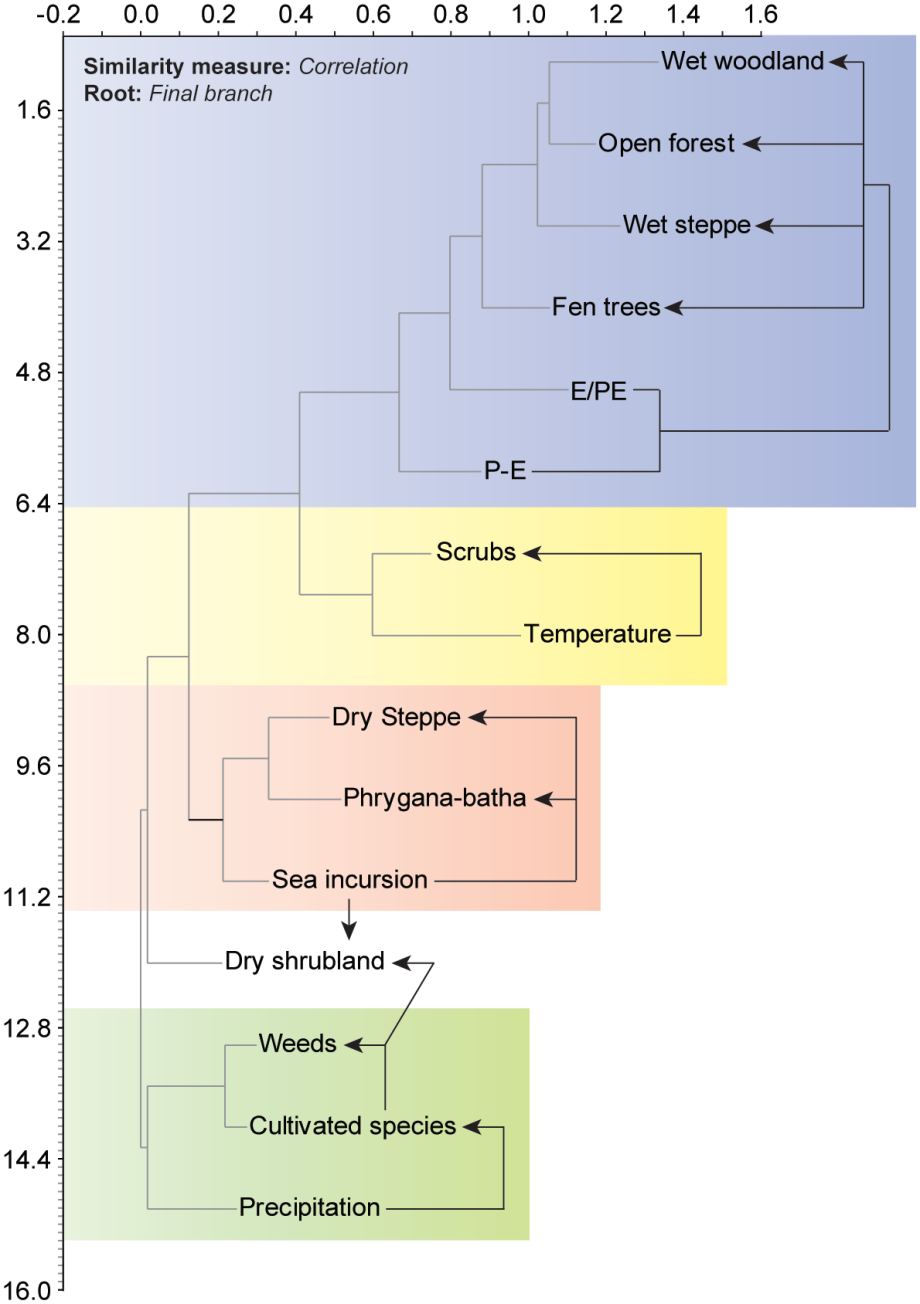
Neighbour Joining analysis of the selected pressures and pollen-derived vegetation patterns. The dendrogram shows the statistical link between one pressure and pollen-derived vegetation patterns, suggesting an environmental relationship between the pressure and the resulting vegetation. Each numerical link between a pressure and a pollen-derived vegetation pattern is denoted by a different colour.

The link between these independent proxies and the ^Pd^Clusters was established using NJ analysis (Fig. 7). This clustering analysis shows the highest affinities between environmental pressures and the ^Pd^Clusters, suggesting a close relationship during the last 6000 years.

### Smoothing

In figures 3 and 5, polynomial smoothing curves were selected to synthesise the main changes observed at the site and in the EM. The best smoothing was chosen according to the highest R^2^.

### Vegetation model

The vegetation model BIOME4 [24] is used in an inverse mode to reconstruct climate along the pollen diagram taking into account variations in the atmospheric concentration of CO_2_ [25]. The reconstruction is based on a Bayesian approach where a priori information on the climate at a given site and time period is refined given the corresponding pollen assemblages and the BIOME4 model, assuming that the differences between model simulations and data follow a Gaussian law. The results are validated using regional modern pollen assemblages. The reconstructions for the Holocene are expressed as anomalies from the estimated modern climate (top core). In this paper, we focus on the ratio of actual and potential evapotranspiration (E/PE), which is an index of drought felt by vegetation, and on the difference between annual precipitation and actual evapotranspiration (P-E), which is an estimate of the site’s contribution to run-off in the catchment basin (Fig. 5 and Table S3). The two other proxies (temperature and precipitations) for the Eastern Mediterranean are derived from Kaniewski *et al.* [19] (Table S4). All reconstructions concern the Eastern Mediterranean (data from Turkey, Cyprus, Syria, and Israel) and are considered as independent proxies.

## Results

The ^P^VD shows that, during the sea incursion deduced from the presence of Dinoflagellate cysts [4000-2900 calibrated year Before the Present (cal yr BP)], the coastal vegetation evolved from a wet woodland through a shrubland to an open-steppe (Fig. 5) similar to those that can be found nowadays along the coast of Akko, western Galilee (Fig. 3). Before the sea incursion, from *c.* 6000 to 4000 cal yr BP, the area was dominated by wet woodland (Fig. 4), with a limited influence of the salt spray brought inland by the northwesterly sea breeze. The relative absence of dinoflagellate cysts suggests no sea incursion and no direct pressures on ecosystems by saline waters. The P-E polynomial curve shows that run-offs were important, yielding higher local freshwater budget/outflows in the EM. Sea-level rise, that started around 8000 cal yr BP in the Haifa Bay and reached the coring site at 4000 cal yr BP, acted like an erosive agent that cumulated with human pressures and climate instability (Fig. 5), leading to an alteration of ecosystems (Fig. 2) and to a shift from a wet woodland to a thorny shrub-steppe (Fig. 5). The sea incursion is directly associated with the development of the ^Pd^Clusters dry steppe and phrygana in the coastal area (Fig. 7). The P-E values indicate a decrease of freshwater budget/outflows, mainly during three periods: 4200-3800, 3500 and 3200-2900 cal yr BP. The E/PE drops after 4000 cal yr BP, reaching its lowest value at 2950 cal yr BP (0.43). Coastal progradation started around 3400-3300 cal yr BP and ended at 2900 cal yr BP at the coring site (disappearance of dinoflagellate cysts) and is concomitant with one of the driest/warmest phases from 3200 to 2900 cal yr BP. Owing to the cumulative pressures, the shrub-steppe turned into an open-steppe and human activity decreased (Fig. 5). The dry wooded components then recovered and dominated until 2100 cal yr BP, mainly led by anthropogenic pressures (Fig. 7). Both steppe and shrubs have remained dominant since 2100 cal yr BP (Fig. 2), and have increased in sub-recent times (Fig. 5), despite more important run-off and a higher local freshwater budget.

## Discussion

A full understanding of the ecological consequences of future relative sea-level variations not only needs to integrate numerous amplifiers (e.g. human impact, urban development and increasing drought), but also to consider the complex mechanisms at work at various timescales [26]. At Akko, the RMAs show that the anthropogenic pressures, with an R^2^ value of 0.81 (Fig. 7), best explain the vegetation dynamics. The high values associated with climate pressures [precipitation (R^2^ = 0.75) and temperature (R^2^ = 0.71)] and sea level changes (R^2^ = 0.70), suggest that these three parameters have interacted over the last 6000 years.

### Sea ingression

The altimetry data of the Topex/Poseidon satellite have shown that sea level rose continuously at a rate of up to 20 mm per yr^−1^ over the period 1993–1999 in the Levantine basin [27]. The predicted maximum value for the year 2100 in the EM is 60 cm higher than the 1990 value, providing an analogue with Haifa Bay where the sea transgressed eastwards (>2.5 km) and flooded the Zevulum Plain (Fig. 1) [28]–[29]. Regardless of the physical mechanisms responsible for the sea ingression on the Zevulum Plain and the local influence of the hydro-sedimentary budget from the Na’aman River [28], the added pressure of the sea invasion between 4000 and 2900 cal yr BP on ecosystem dynamics already affected by human activity and climate stress, led to a deep ecological erosion (Fig. 5).

The main consequences of the sea invasion were a retreat of the coastal forest (drop in forested/wooded ^Pd^Clusters) (Fig. 5), a loss of resilience and a disappearance of the initial local biogeographic zonation. The forest replacement by a thorny shrub-steppe and then by an open-steppe appeared to follow, rather than cause, failure of tree regeneration. To sum up, several causes cumulatively mediated forest dynamics between 4000 and 2900 cal yr BP: (i) the lack of regenerating stands of different tree species responsible for the retreat of the coastal forest [26], [30]–[31] which probably resulted from the increases in saltwater intrusions, and coastal erosion that washed away entire tree stands and provoked seedling burial by shifting sands along the sandy coast of Haifa Bay [18]; (ii) changes of the physical characteristics of the pedologic substrate following coastal progradation, with increasing salt concentration and sand deposition in the Bay; and (iii) increased human pressures and climate forcing [19].

### Exposure to salt

**Figure 8:**
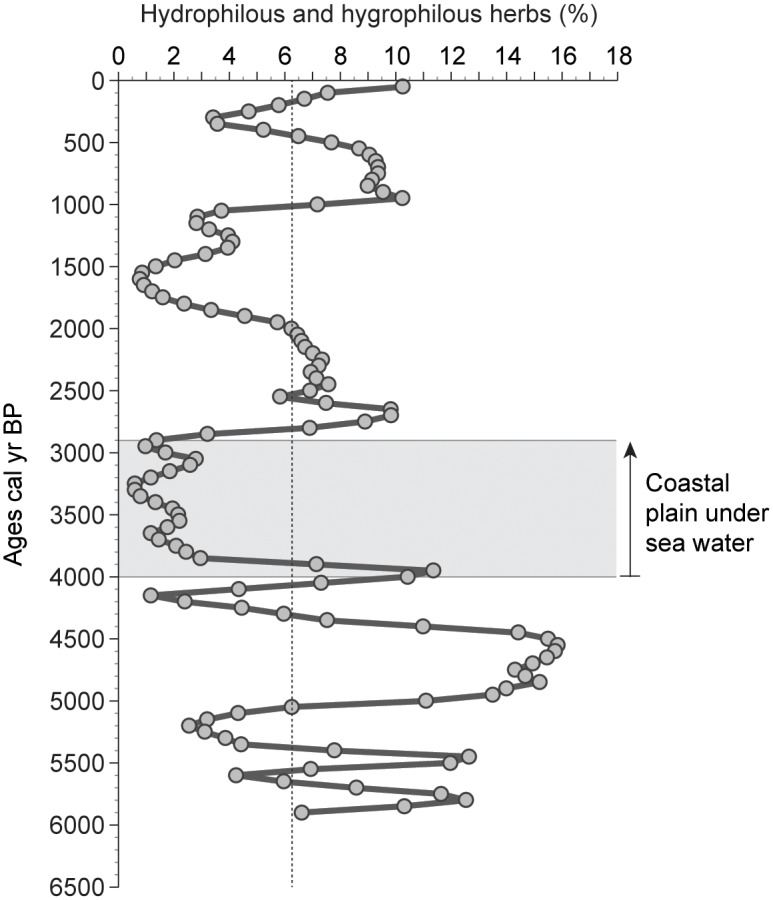
Hygrophilous and hydrophilous herbs from Haifa Bay. The hygrophilous and hydrophilous herbs, expressed as percentages, are displayed as a single curve plotted on a linear timescale. The average value is indicated by a vertical dotted line. The maximum sea ingression is bracketed between the two lines.

Due to the intrusion of the saline water table in fresh water streams, the freshwater wetland associated with the Na’aman River was deeply impacted after 4000 cal yr BP, with a fall in hygrophilous-hydrophilous herbs (Fig. 8). The subsequent coastal progradation that started at 3400-3300 cal yr BP (Fig. 5) left an eroded sandy-salty area, colonized by a steppic vegetation that became dominant until the end of the shoreline retreat (2900 cal yr BP). A similar process was observed in the Salt Lake of Larnaca, Cyprus, where a shift from sheltered marine to lagoonal environments between 3400 and 3300 cal yr BP produced an ecological change with a strong increase in xerophytic vegetation-types colonizing the shores that were no longer washed by seawater [32].

### The end of the sea incursion

During the marine stage at Akko, the main impact on ecosystems was identified at 3200 cal yr BP, when the physical processes linked with the regression of the sea were added to higher climate pressures, leading to decreased human activities (Fig. 5). The 3.2 kyr BP drought event is now well identified in different locations from Syria [33]–[35], Cyprus [32], Israel [36]–[41], and Egypt [42]. The signature of this climate event is particularly strong at Akko because the effects of drought were reinforced by the physical processes linked to the coastal changes, causing a dramatic demise in wooded ecosystems (Fig. 5). After 4000 cal yr BP, the wet woodland, the open-forest, and the fen trees never regained their former importance in the coastal area. The wooded components recovered after 2850 cal yr BP, but only dry shrubland (Fig. 4) was able to develop, until 2100 cal yr BP (Fig. 5).

### Coastal retreat

Shoreline migration analyses of the coast of Akko, northern Haifa Bay, during the last 200 years, show that the coast retreated by 15 meters from 1926 to 2006, primarily due to human-induced modification of the Na’aman River’s hydro-sedimentary budget, changes in sediment transport, and coastal structures built during the last 50 years [43]. This new phase of sea-level invasion is well attested by high values of steppic vegetation (Fig. 5) and a renewed drop in hygrophilous-hydrophilous herbs (Fig. 8) at the very end of the sequence. This suggests that similar stresses generate analogous biological processes, whatever the period under consideration.

### Clues for the future

The long-term environmental evolution of the northern part of Haifa Bay suggests that the future of coastal ecosystems and wetlands in the southern Levant will mainly be mediated by complex interactions between human impacts, climate change and changing coastal geomorphology. This questions human behavior in these fragile coastal systems: how will humans respond to rising relative sea level and increased drought, and how will they produce a sustainable management of coastal ecosystems and wetlands while their encroachment already affects the landscape, through an overexploitation of resources and by an overuse of freshwater. In Haifa Bay, human activities have played an important role during the last 4000 years, by reinforcing and gradually supplanting the natural stressors. Efficient management of the coastal habitats of southern Levant would imply a strong reduction in anthropogenic pressures linked to urban development, in order to mitigate the predicted effects of Global Change. Modern cities in the Levant are undergoing strong demographic growth, but their ongoing expansion needs to carefully assess the environmental limits of local natural resources and ecosystems’ capacity to respond.

## Conclusions

Haifa Bay’s biogeographical evolution at millennial- to centennial time-scales suggests that projected sea-level rise scenarios for the next century will deeply impact the coastal ecosystems, especially within the context of more intense drought stress, increased human activities and diminished sediment supply by local rivers. The major physical processes that have shaped the ecosystems since 6000 cal yr BP can be attributed to climate fluctuations (Fig. 6) and to the sea-level transgression 4000 cal yr BP and subsequent coastal retreat since 3400-3300 cal yr BP. Stand-level regeneration failures of the forest-woodland vegetation, as well as a high rate of freshwater wetland loss, might be expected in the coastal southern Levant in the near future.

## Supporting Information

Table S1 **Data - Modern pollen samples.**
(XLSX)

Table S2 **Data - Samples from the core.**
(XLSX)

Table S3 **Data - Reconstructed climate values for the Eastern Mediterranean, E/PE and P–E.**
(XLSX)

Table S4 **Data - Reconstructed climate values for the Eastern Mediterranean, annual precipitation, mean temperature of the coldest month, mean annual temperature, mean temperature of the warmest month.**
(XLSX)
